# OpenOR – a virtual reality framework for medical education

**DOI:** 10.1007/s00701-026-06968-3

**Published:** 2026-07-01

**Authors:** Florian Grimm, Brenno Santana Tagliavini, Felix Behling, Marcos Tatagiba, Borris Golinski, Patrick Haas, Isabel Gugel, Constantin Roder

**Affiliations:** Department of Neurosurgery, University Hospital Tübingen, Hoppe-Seyler-Strasse 3, 72076 Tübingen, Germany

**Keywords:** Virtual Reality, Education, Neuroanatomy, Neurosurgery

## Abstract

**Purpose:**

Virtual reality (VR) is increasingly recognized as a powerful tool in medical training. It offers immersive, interactive environments ideal for exploring complex anatomy and procedures. With the advent of affordable hardware, VR can provide high-quality, cost-effective training experiences.

**Methods:**

We developed the VR application OpenOR in Unity for medical education. It supports multi-user interaction with customizable avatars and hand-tracking for intuitive control. Designed for medical students and professionals, OpenOR enables the exploration of complete clinical case studies. This includes medical history, a virtual DICOM viewer, visualization of anatomical structures and pathologies derived from MRI segmentations, and the original 3D surgical videos for each case. In addition, the 3D MRI-based models are complemented by CAD (computer-aided design) models of real implants, such as VP shunts or aneurysm clips. A usability test was conducted with 30 medical students using structured questionnaires.

**Results:**

The pilot implementation demonstrated OpenOR’s educational potential. Students reported increased engagement and a perceived improvement in understanding of anatomy and surgical workflows. The multi-user mode fostered collaborative learning, while hand-tracking supported intuitive interaction. The combination of virtual PACS viewing, interactive 3D models, and surgical 3D videos provided a realistic and integrated learning experience. The evaluation feedback indicated high user satisfaction and confirmed the platform's value for neurosurgical and anatomical training.

**Conclusion:**

OpenOR represents a significant advancement in VR-based medical education. By integrating immersive technologies with affordable hardware, it offers practical and scalable training solutions for Neurosurgery and Neuroanatomy.

## Introduction

Virtual Reality (VR) is a rapidly evolving technology increasingly used across various medical fields. These include medical training and patient care, where a shift from non-immersive, screen-based simulations [9] to fully immersive experiences with head-mounted displays (HMDs) and new interaction methods is evident [18]. The availability of high-performance, consumer-grade VR hardware has made high-quality medical simulations more accessible and scalable [17].

Technological advancements have significantly enhanced the capabilities of immersive VR systems, enabling the real-time rendering of highly detailed, three-dimensional (3D) models with complex geometries at high resolution [13]. New interaction methods, such as hand tracking, enable intuitive and natural user engagement with virtual environments, reducing the need for extensive familiarization or training. Additionally, VR platforms are increasingly adding multi-user features and real-time collaborative tools, creating engaging and interactive educational environments [18].

These advances enable more effective visualization of complex 3D anatomical structures [1] and clinical procedures [3], offering medical students and healthcare professionals realistic, high-fidelity learning experiences [11, 21, 28]. Multiple meta-analyses and systematic reviews have demonstrated that VR-based interventions can enhance knowledge acquisition, spatial understanding, and procedural skills in medical education [17]. Furthermore, VR enhances learner engagement and facilitates realistic, case-based training scenarios — including patient interactions and emergency management — without the ethical, logistical, and safety issues associated with making first experiences in the actual clinical setting [16, 18, 28]. Furthermore, this technology enables a playful approach to complex clinical problem-solving.

Additionally, the increasing use of preoperative imaging, intraoperative 3D recording, and virtual anatomical reconstructions underscores the growing importance of VR in this field.

Among medical specialties, neurosurgery is a highly intricate and evolving field that greatly benefits from VR-based educational tools [7, 25]. Neurosurgery places particularly high demands on spatial cognition, anatomical precision, and the interpretation of complex imaging data. Many of the structures relevant to neurosurgical decision-making—such as neurovascular relationships, skull base anatomy, and deep-seated lesions—are difficult to conceptualize using conventional two-dimensional images or static atlases. The detailed spatial relationships of neuroanatomical structures offer unique opportunities for immersive technologies to improve understanding of complex neurosurgical anatomy and procedures. Against this background, virtual reality (VR) has gained increasing attention as a complementary tool for neurosurgical education, training, and clinical preparation [6, 23].

Immersive VR environments allow three-dimensional visualization of patient-specific anatomy derived from magnetic resonance imaging and computed tomography. By enabling users to explore and manipulate these reconstructions interactively, VR may facilitate spatial orientation and a more intuitive understanding of complex anatomical relationships. Several studies have reported higher engagement and improved perceived understanding of neuroanatomy when VR-based approaches are incorporated into teaching, particularly in anatomically challenging regions such as the skull base [24] and vascular pathologies [8].

In addition to anatomical education, VR has been applied to procedural training and surgical simulation in neurosurgery. A variety of simulators have been developed for tasks such as ventricular catheter placement, endoscopic skull-base approaches, tumor resection, and aneurysm clipping [4, 5, 22]. These systems offer the opportunity for repeated practice in a controlled, risk-free setting and may support the acquisition of technical skills, familiarity with operative workflows, and hand–eye coordination.

VR has also been explored as a tool for preoperative planning and case discussion. Patient-specific immersive models have been used to visualize lesion extent, vascular anatomy, and potential surgical corridors, thereby supporting the development of operative strategies and interdisciplinary communication [26]. In particular, immersive visualization has been reported to enhance spatial awareness and surgeon confidence when planning complex skull base or vascular cases [15, 30]. Collaborative and multi-user VR environments have emerged as an extension of these applications. Such platforms allow multiple users to share a virtual space, manipulate anatomical models together, review imaging, and discuss operative strategies in real time [12, 26]. For educational purposes, the first multi-user collaborative spaces with photogrammetric models were introduced, yielding promising results and enabling direct interaction between teachers and students [7].

Current VR-based educational applications in medicine often emphasize specific content elements, such as anatomical models, surgical videos, or isolated 3D visualizations. While these approaches provide valuable learning opportunities, more integrative, interactive learning environments that combine multiple data modalities and support collaborative exploration remain relatively uncommon.

To explore this direction, OpenOR has been created as an innovative virtual reality (VR) application for neurosurgical education. Built on the Unity engine, OpenOR features interactive 3D visuals, case-based learning, multi-user collaboration, and advanced interaction techniques. The platform combines various types of medical data, including 3D surgical videos, text-based case descriptions, patient-specific 2D Digital Imaging and Communications in Medicine (DICOM) MRI datasets, CAD models of medical implants, and high-resolution 3D reconstructions of anatomical structures and clinical environments.

This immersive environment allows users to explore complex neurosurgical case scenarios, interactively navigate surgical 3D videos, and examine the spatial relationships of anatomical structures using patient-specific imaging data. Additionally, OpenOR offers multi-user functionality with avatar-based representation and integrated voice-over-IP (VoIP), enabling organized group training sessions within the virtual environment over the internet.

This study aims to introduce the initial implementation of OpenOR and to evaluate its feasibility, usability, and educational potential through a pilot project involving medical students.

## Methods

### App-design

The application was developed using Unity 2023.2.20f1 (www.unity.com). The Meta XR Core Software Development Kit (SDK) (developers.meta.com) was utilized for technical integration and deployment of visualization. User interaction within the VR app is supported by the Meta XR Interaction SDK for Unity, which enables native hand tracking. This allows users to manipulate objects, including grabbing, poking, snapping, and teleporting, resulting in an intuitive and immersive experience.

The Meta Avatars SDK was used to visualize individual networked avatars. Network synchronization of motion data, networked objects, and Voice over IP (VoIP) communication is managed through Fusion Meta XR Integration (www.photonengine.com), ensuring real-time, multi-user interaction within the virtual environment.

### Virtual environment

**Fig. 1:**
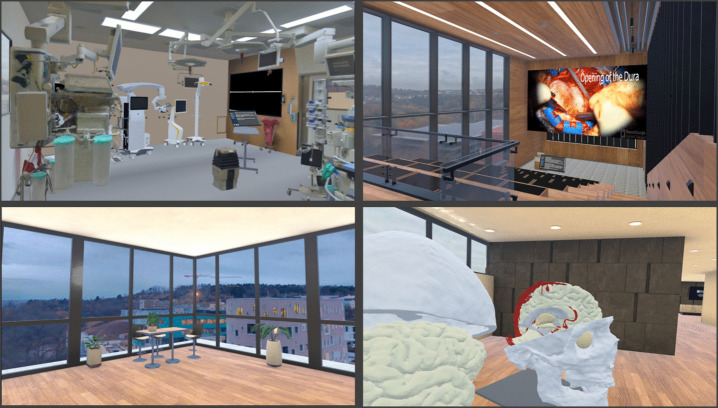
Virtual environment showcasing a surgical operating theatre, a meeting room, and an auditorium to support immersive medical training and collaborative learning

The 3D environment was created using a photogrammetric reconstruction of an operating room [21], combined with several commercially available prefabs representing the clinical complex and auditory features for teaching. The addition of CAD models of medical devices further enhances visualization. With support from the manufacturers, the application features a Curve® Navigation system (Brainlab, Munich, Germany) and a Kinevo surgical microscope (Carl Zeiss Meditec AG, Jena, Germany), providing realistic and interactive views of essential neurosurgical equipment. As a skybox background, a 360° panoramic photograph of the University Hospital complex enhances realism and spatial immersion (Fig. 1).

### Case scenarios

For the initial evaluation, five neurosurgical case scenarios were incorporated into the VR application:

1. Clinoidal meningioma with resection and decompression of the optic nerve canal.
2. Tuberculum sellae meningioma with hydrocephalus after ventriculoperitoneal shunt placement and tumor resection.
3. Middle cerebral artery aneurysm with microsurgical clipping.
4. Vestibular Schwannoma: tumor resection via a retrosigmoid approach in a semi-sitting position.
5. Large petroclival meningioma: tumor resection via a retrosigmoid approach in a semi-sitting position.

After obtaining written informed consent for data use, anonymized patient data were collected for the selected cases, including medical history, clinical examinations, preoperative imaging (MRI, CT, angiography), and stereoscopic 3D surgical video recordings. Ethical approval was not required for this work, as it did not involve a patient study.

Three-dimensional models were created by segmenting preoperative T1-weighted, contrast-enhanced MRI scans (thin-slice data). Anatomical structures were semi-automatically segmented using Elements (Brainlab, Munich, Germany), while tumor segmentation was performed manually. Based on the segmented MRI data, CAD STL (Surface Tessellation Language) files were generated and imported into Blender 4.2 (www.blender.org) for further refinement. In Blender, polygon reduction and surface smoothing were applied to optimize the anatomical models for real-time visualization.

**Fig. 2:**
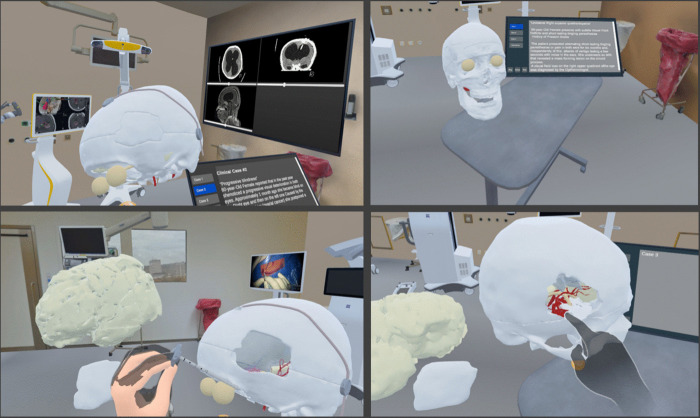
Interactive case-based learning environment displaying 3D anatomical models, case descriptions, an integrated MRI/CT viewer, and CAD models of medical implants for improved visualization and interaction

Medical implants such as the ventriculoperitoneal (VP) shunt system (proGAV® 2.0, Christoph Miethke GmbH & Co. KG, Potsdam, Germany) and the aneurysm clip (L-Aneurysm Clip®, Peter Lazic GmbH Microsurgical Innovations, Tuttlingen, Germany) were placed orthotopically within the anatomical models (Fig. 2).

The final CAD models were then exported for integration into Unity, where they were enhanced with snap-interaction features, scalable interactions, network capabilities, and standardized textures representing various anatomical modalities. This process ensures the development of interactive, high-fidelity representations of patient-specific anatomy and surgical procedures within the virtual reality (VR) environment.

Within the application, users can select cases using an interactive touchscreen. When a case is selected, a 3D anatomical model generated from MRI segmentation appears, along with the case description, 2D imaging data in the DICOM viewer, and a 3D video.

After reviewing the case description, students can analyze the available 2D images in the DICOM viewer and discuss the pathology, anatomical relations, and possible surgical approaches. Afterwards, the clinical case can be addressed using the immersive 3D MRI CAD model of the skull, with the option to open the skull via a pre-planned craniotomy and manipulate various anatomical structures, including the relevant pathology. Afterwards, the case-specific stereoscopic 3D surgical video, cut to approximately 5 min, will be viewed on a virtual display in the operating room (OR) or on a cinema-scaled screen in the virtual auditorium (see also Video 1: https://vr-video.neurochirurgie-tuebingen.de).

All elements within the application are synchronized through the Internet. This ensures that multiple users can interact with and view case data simultaneously in real time, promoting a collaborative, interactive training environment.

### User testing and experience

**Fig. 3:**
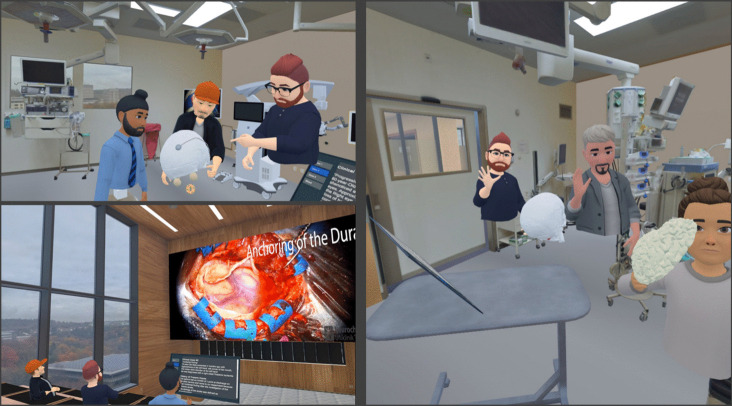
Interactive teaching scenario with networked avatar visualization and real-time manipulation of networked objects, supporting collaborative learning in a multi-user VR environment

All cases were reviewed with medical students. The sessions were held in groups of 2–5 students, supervised by an experienced neurosurgeon. All participants joined training sessions in the VR application as networked avatars, enabling real-time interaction, collaborative case discussions, and synchronized exploration of anatomical models and imaging data (Fig. 3). This multi-user feature promoted immersive peer-to-peer learning and instructor-led guidance, enhancing the educational experience. Each group worked through two to three case scenarios in a structured session lasting 45–60 min.

**Fig. 4:**
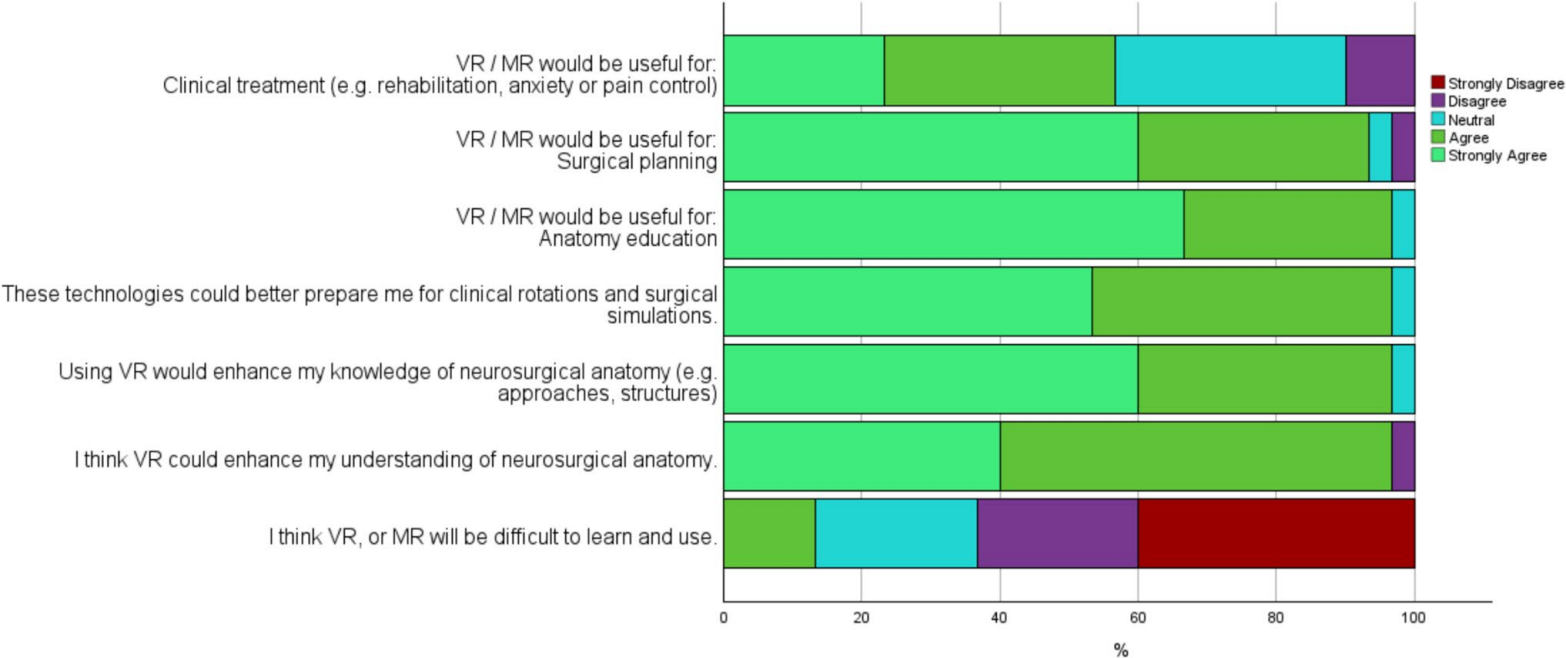
Distribution of the answers to general attitudes concerning VR techniques in medicine and medical education (*n* = 30 students)

**Fig. 5:**
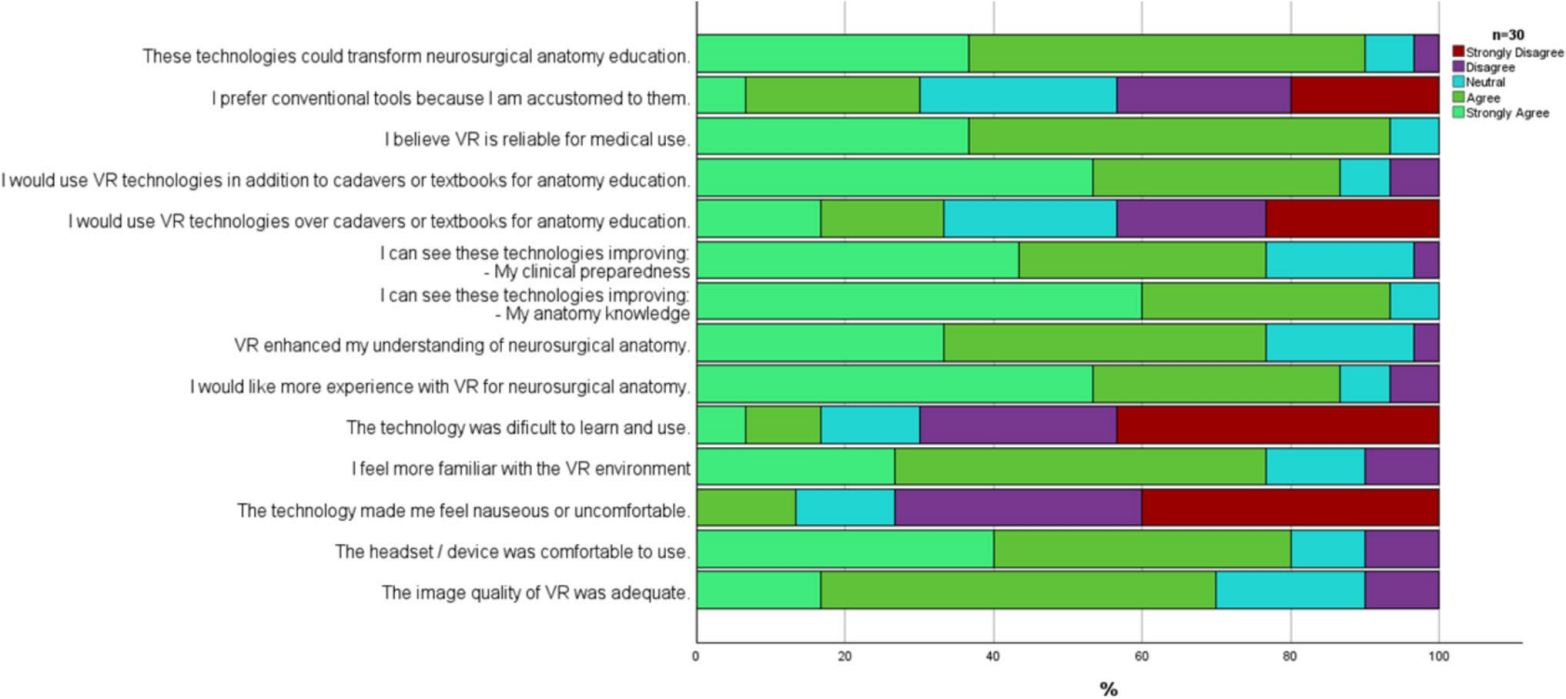
App-specific distribution of the answers after undergoing the VR training (*n* = 30 students)

After the VR-based training, the learning outcomes and user experience were assessed using standardized 22-point questionnaires (Figs. 4 and 5). The questionnaire was an exploratory, non-validated instrument developed for this pilot study. It assessed perceived usability, engagement, subjective learning experience, and user comfort/cybersickness using 5-point Likert scales. No formal assessment of internal consistency or construct validity was performed; therefore, the results should be interpreted as descriptive and exploratory. This enabled the first evaluation of the application's educational effectiveness and usability.

### Statistical evaluation

SPSS Version 29 (IBM) was used for statistical evaluation. Data was not normally distributed as assessed by the Shapiro–Wilk test. Therefore, a Mann–Whitney U test was conducted to assess differences in questionnaire responses across sex, prior VR experience, visual impairment, and gaming experience.

## Results

The initial deployment of OpenOR demonstrated its potential as an effective educational tool. Users reported high engagement and a perceived improvement in understanding of anatomical structures and surgical workflows. The multi-user feature promoted collaborative learning, while hand-tracking allowed for natural interaction within the virtual environment. Case-based training, combined with a virtual PACS viewer, case descriptions, and supernaturally immersive 3D CAD models, enables realistic theoretical teaching. This can be connected to practical knowledge by watching the 3D stereoscopic surgical microscope recordings of the cases in a virtual auditorium with a virtual screen larger than 100 m^2^.

All students completed the whole course in VR, and no significant adverse events occurred that limited the training. Four participants reported mild discomfort or nausea during the VR session; none discontinued the training.

General data for the participants were: n = 30; females = 18; average age = 21.4 years ± 3.2 years. 18 reported using VR techniques before, 12 reported visual impairments such as myopia, hyperopia, or astigmatism, and 17 had played video games previously.

The results of the questionnaires are shown in Figs. 4 and 5 and in Table 1.

**Table 1:** Questionnaire items and corresponding answers (median, mode, range)

Item	Median	Mode	Range
VR/MR would be useful for: Clinical treatment (e.g. rehabilitation, anxiety, or pain control)	4,00	3^a^	3
VR/MR would be useful for: Surgical planning	5,00	5	3
VR/MR would be useful for: Anatomy education	5,00	5	2
These technologies could better prepare me for clinical rotations and surgical simulations	5,00	5	2
Using VR would enhance my knowledge of neurosurgical anatomy (e.g., approaches, structures)	5,00	5	2
I think VR could enhance my understanding of neurosurgical anatomy	4,00	4	2
I think VR, or MR will be difficult to learn and use (pre-training)	2,00	1	3
These technologies could transform neurosurgical anatomy education	4,00	4	3
I prefer conventional tools because I am accustomed to them	3,00	3	4
I believe VR/MR is reliable for medical use	4,00	4	2
I would use VR technologies in addition to cadavers or textbooks for anatomy education	5,00	5	3
I would use AR/VR/MR technologies over cadavers or textbooks for anatomy education	3,00	1^a^	4
I can see these technologies improving: My clinical preparedness	4,00	5	3
I can see these technologies improving: My anatomy knowledge	5,00	5	2
VR enhanced my understanding of neurosurgical anatomy	4,00	4	3
I would like more experience with AR/VR/MR for neurosurgical anatomy	5,00	5	3
The technology was difficult to learn and use. (post-training)	2,00	1	4
I feel more familiar with the VR environment	4,00	4	3
The technology made me feel nauseous or uncomfortable	2,00	1	3
The headset/device was comfortable to use	4,00	4^a^	3
The image quality of VR was adequate	4,00	4	3

Strongly Disagree (1), Disagree (2), Neutral (3), Agree (4), Strongly Agree (5). If multiple modes are available, the smallest value is displayed (a)

### Statistical group differences

The distribution of the answers regarding sex, visual impairment, and gaming experience did not reveal a different distribution within groups.

Between students who reported prior VR experience and those without any VR experience, there was a significant difference in the point “The technology made me feel nauseous or uncomfortable”. Across experiences, the median response was 1.5 (totally disagree to disagree) and 2.5 (disagree to neutral), indicating slightly lower comfort among inexperienced users (U = 59, z = −2.19, p = 0.039). In this group, consent to use VR technologies in addition to cadavers or textbooks for anatomy education was slightly lower for inexperienced users (median: 4, “agree”) than for experienced users (median: 5, “totally agree”; U = 157, z = 2.3, *p* = 0.039).

## Discussion

The integration of Virtual Reality (VR) into medical education offers significant advantages, as demonstrated by the presented development. Complex three-dimensional anatomical relationships, along with the spatial arrangement of implants and surgical instruments, can be realistically visualized within a virtual environment [10, 14, 20]. This immersive visualization is crucial for developing a profound spatial understanding and enhancing visualization skills. For example, systematic reviews and meta-analyses [17, 19, 31] have shown that VR and Augmented Reality (AR) can improve test performance in medical and science students regarding physiology and anatomy. The application and evaluation of virtual technologies in anatomical education, as well as surgical views on anatomy, are particularly emphasized [27]. This is also reflected in our results. In our cohort, most students reported the perception of improvement in anatomical understanding of complex neuroanatomy after using the app and working through the cases (Agree—Strongly Agree, 93.3%). Overall, most participants consider VR an additional tool alongside traditional education methods, including cadaver studies (Agree—Strongly Agree 86.8%), and most express a desire to participate in more VR courses (86.6%).

The combination of hand tracking and natural object manipulation further enhances the learning experience by allowing users to interact with and manipulate virtual objects in a natural, intuitive way. This enables users to literally "grasp" spatial relationships in a way that is difficult to achieve with traditional 2D images or static 3D models, significantly boosting the authenticity and effectiveness of the training [17, 29]. As a result, the system's learning curve is surprisingly short, encouraging quick user adoption and fostering a natural, immersive learning environment. This is reflected in the fact that most users find the app easy to use (Agree—Strongly Agree 70%).

Additionally, synergistic effects were observed through interactive group teaching sessions in the virtual environment. The combination of avatar-based representation, gesture-based interaction, manipulation of 3D anatomical models, and direct voice communication helped create a highly natural and collaborative learning environment. These social and interactive elements appear to enhance the platform's educational value beyond individual study, fostering a more immersive, socially engaging learning experience.

Compared with many currently available VR-based educational systems in neurosurgery, OpenOR is characterized more by the integration of multiple complementary data types within a single collaborative environment than by a focus on isolated training tasks [6, 23]. While existing platforms often focus on either anatomical visualization, procedural simulation, or surgical video review, OpenOR combines interactive three-dimensional anatomy, patient-specific imaging, stereoscopic surgical videos, and real-time multi-user interaction in a single shared virtual space. This integrative approach enables structured, case-based discussion that more closely reflects clinical teaching workflows in neurosurgery. The collaborative multiuser elements, which enable direct virtual interaction between users, seem to add value to VR applications [7, 12, 26]. The multi-user design allows instructors and learners to explore cases simultaneously, supporting guided teaching and peer interaction rather than individual, self-directed use. From a technical perspective, the reliance on hand tracking and teleportation-based navigation reduces interaction complexity and may lower the entry barrier for VR-naïve users. While these characteristics do not, in themselves, demonstrate superior educational effectiveness, they suggest practical advantages of OpenOR as a teaching and discussion platform, particularly in supervised small-group settings.

It is important to note that this represents a new approach in medical education. The initial high levels of engagement and enthusiasm for VR-based visualization might be driven by the novelty of the technology and the unfamiliarity of immersive environments in traditional educational settings. Whether this acceptance and enthusiasm can be maintained over time remains uncertain and calls for long-term studies, especially given the current lack of standardized, validated guidelines for VR training, which hinders the production of high-quality evidence [14, 17].

Another important aspect concerns accessibility and user comfort. Overall, the VR sessions could be completed by all participants; however, a subset of users reported mild adverse effects. Four students experienced transient discomfort or nausea during the training, a phenomenon commonly associated with immersive VR applications using head-mounted displays [2]. No participant discontinued the session because of these symptoms.

The relatively low incidence and mild nature of these effects may be related to the design of the virtual environment, which relies on stable visual references and avoids continuous artificial motion. Interaction was primarily based on hand tracking and teleportation-based navigation, both of which are considered less disorienting than smooth locomotion. Technical factors such as reduced latency, higher frame rates, and stable backgrounds have previously been shown to mitigate cybersickness [17] and were incorporated into the system design.

Notably, all participants who reported discomfort had no prior VR experience, whereas users with prior VR exposure reported significantly fewer symptoms. This observation suggests a potential habituation effect, as described in prior VR studies. Nevertheless, given the limited sample size, these findings should be interpreted cautiously and regarded as descriptive rather than conclusive.

The current cost of acquiring VR headsets and compatible hardware has dropped significantly in recent years, making it feasible to equip small groups of students or trainees for collaborative learning sessions. Although the cost of consumer-grade VR hardware has decreased in recent years, affordability and scalability remain highly context-dependent and vary across institutions, regions, and implementation models. While financial considerations are often seen as a barrier to VR adoption, the long-term cost-effectiveness relative to traditional methods (e.g., cadaver-based training) can be substantial, making VR an increasingly practical option [14]. Nevertheless, the current VR training setup is best suited for small, supervised student groups. Scaling the experience to accommodate whole cohorts or entire semesters remains challenging due to logistical and technical limitations.

The platform is designed to be scalable and modular, making it easy to add more clinical cases and support the ongoing growth of the case library. Additionally, the central concept might be expanded to other medical fields beyond neurosurgery, such as preclinical anatomy, neurology, other surgical disciplines, or even radiology, where spatial awareness and procedural planning are equally important. Modularity enhances the versatility of VR in medical education, enabling a range of applications across specialties, including surgical procedures, emergency scenarios, and non-technical skills training.

With increased development resources, it would be possible to simulate more complex procedural workflows, including interactive training modules for tasks such as ventriculoperitoneal shunt placement, external ventricular drain (EVD) insertion, or craniotomy and patient positioning planning. Given the current capabilities of modern VR systems, the technological limitations in both hardware and software are minimal, providing significant flexibility for future innovations in this area.

While OpenOR shows promising results for improving neurosurgical and anatomical education, several limitations still exist. The technology's novelty might generate initial excitement, but it also raises concerns about maintaining long-term user engagement. This study lacked a control group, limiting the extent to which the findings can be generalized. Including a control group in such a small, specialized educational setting poses significant challenges. Although most users tolerated the VR experience well, some—especially those new to VR—reported discomfort, highlighting the need for thorough onboarding and more research into user comfort. Additionally, even though hardware costs have decreased, wider adoption still faces logistical and financial obstacles.

## Limitations

Several limitations of this study should be acknowledged. First, the work was conceived as a pilot evaluation of the in-house-developed app, focusing on feasibility and usability, as reflected in the relatively small sample size. Both the overall cohort and the subgroups used for exploratory analyses were limited, reducing statistical robustness and precluding firm conclusions from subgroup comparisons.

Second, the study design did not include a control group, pre–post assessment, or objective measures of knowledge or learning effect. As a result, the findings are limited to subjective user reports and do not support conclusions about actual learning gains or performance improvements. Any statements related to educational value should therefore be interpreted as perceived rather than objectively demonstrated effects.

Third, user experience was assessed using a study-specific questionnaire that had not been formally validated. While the instrument was suitable for exploratory purposes, no analyses of internal consistency or construct validity were performed, which limits the interpretability and generalizability of the results.

In addition, the statistical analyses were exploratory. Multiple subgroup comparisons were performed without correcting for multiple comparisons, increasing the likelihood of a type I error. Reported p-values should therefore be interpreted cautiously and viewed as hypothesis-generating rather than confirmatory.

Finally, novelty effects and differences in prior experience with VR technology may have influenced participant responses. Although training sessions were standardized, varying levels of familiarity with immersive systems could have affected both usability ratings and perceived educational benefit.

Taken together, these limitations indicate that the present study primarily demonstrates the technical feasibility and user acceptance of the OpenOR platform. Larger studies incorporating validated assessment tools, objective outcome measures, and controlled designs will be necessary to more rigorously evaluate its educational impact.

## Conclusion

The OpenOR app is a promising platform for medical training for students and a solid concept that could be expanded into neurosurgery, neuroanatomy, and potentially other subjects. Multimodal cases, CAD models, and surgical videos can be explored in an immersive, collaborative environment during group lessons. Students generally received the app well and found it intuitive to use. It could represent an addition to the teaching spectrum alongside traditional methods.

## Data Availability

Data are available upon reasonable request by contacting the corresponding author.
